# Autism spectrum disorder symptom expression in individuals with 3q29 deletion syndrome

**DOI:** 10.1186/s13229-022-00533-2

**Published:** 2022-12-24

**Authors:** Rebecca M. Pollak, Jordan E. Pincus, T. Lindsey Burrell, Joseph F. Cubells, Cheryl Klaiman, Melissa M. Murphy, Celine A. Saulnier, Elaine F. Walker, Stormi Pulver White, Jennifer G. Mulle

**Affiliations:** 1 Center for Advanced Biotechnology and Medicine, Robert Wood Johnson Medical School, Rutgers University, Piscataway, NJ USA; 2 Department of Pediatrics, School of Medicine, Emory University, Atlanta, GA USA; 3 Marcus Autism Center, Children’s Healthcare of Atlanta and Emory University School of Medicine, Atlanta, GA USA; 4 Clinical Psychology, College of Arts and Sciences, Georgia State University, Atlanta, GA USA; 5 Department of Human Genetics, School of Medicine, Emory University, Atlanta, GA USA; 6 Department of Psychiatry and Behavioral Science, School of Medicine, Emory University, Atlanta, GA USA; 7 Neurodevelopmental Assessment and Consulting Services, Decatur, GA USA; 8 Department of Psychology, Emory University, Atlanta, GA USA; 9 Department of Psychiatry, Robert Wood Johnson Medical School, Rutgers University, 679 Hoes Lane West, NJ 08854 Piscataway, USA

**Keywords:** 3q29 deletion, Autism, Copy number variants, Developmental delay, Genomic disorder, Psychiatric genetics, ADOS-2, ADI-R

## Abstract

**Background:**

The 1.6 Mb 3q29 deletion is associated with neurodevelopmental and neuropsychiatric phenotypes, including a 19-fold increased risk for autism spectrum disorder (ASD). Previous work by our team identified elevated social disability in this population via parent-report questionnaires. However, clinical features of ASD in this population have not been explored in detail.

**Methods:**

Thirty-one individuals with 3q29 deletion syndrome (3q29del, 61.3% male) were evaluated using two gold-standard clinical ASD evaluations: the Autism Diagnostic Observation Schedule, Second Edition (ADOS-2), and the Autism Diagnostic Interview, Revised (ADI-R). Four matched comparators for each subject were ascertained from the National Database for Autism Research. Item-level scores on the ADOS-2 and ADI-R were compared between subjects with 3q29del and matched comparators.

**Results:**

Subjects with 3q29del and no ASD (3q29del-ASD) had greater evidence of social disability compared to typically developing (TD) comparison subjects across the ADOS-2. Subjects with 3q29del and ASD (3q29del + ASD) were largely indistinguishable from non-syndromic ASD (nsASD) subjects on the ADOS-2. 3q29del + ASD performed significantly better on social communication on the ADI-R than nsASD (3q29 + ASD mean = 11.36; nsASD mean = 15.70; *p* = 0.01), and this was driven by reduced deficits in nonverbal communication (3q29 + ASD mean = 1.73; nsASD mean = 3.63; *p* = 0.03). 3q29del + ASD reported significantly later age at the first two-word phrase compared to nsASD (3q29del + ASD mean = 43.89 months; nsASD mean = 37.86 months; *p* = 0.01). However, speech delay was not related to improved nonverbal communication in 3q29del + ASD.

**Limitations:**

There were not enough TD comparators with ADI-R data in NDAR to include in the present analysis. Additionally, our relatively small sample size made it difficult to assess race and ethnicity effects.

**Conclusions:**

3q29del is associated with significant social disability, irrespective of ASD diagnosis. 3q29del + ASD have similar levels of social disability to nsASD, while 3q29del-ASD have significantly increased social disability compared to TD individuals. However, social communication is reasonably well preserved in 3q29del + ASD relative to nsASD. It is critical that verbal ability and social disability be examined separately in this population to ensure equal access to ASD and social skills evaluations and services.

**Supplementary Information:**

The online version contains supplementary material available at 10.1186/s13229-022-00533-2.

## Background

The 3q29 deletion is a rare (~ 1 in 30,000) [1, 2] 1.6 Mb typically de novo deletion on chromosome 3 (hg19, chr3:195725000–197350000) [3–5]. The 3q29 deletion has well-established links to multiple neurodevelopmental and neuropsychiatric disorders, including a 19-fold increased risk for autism spectrum disorder (ASD) [6–8] and a > 40-fold increased risk for schizophrenia (SZ) [9–13]. Individuals with 3q29 deletion syndrome (3q29del) also may have mild to moderate intellectual disability (ID), global developmental delay (GDD), and delayed speech [3–5, 14–21]. Recent efforts have led to an improved understanding of 3q29del, but nuances of some phenotypes, including social disability, require further exploration.

Existing case reports support a link between 3q29del and social disability [3, 4, 14, 18–22]. Additionally, previous work by our team showed that individuals with 3q29del have increased social disability relative to typically developing (TD) controls, independent of whether they meet diagnostic criteria for ASD [6]. Parents of individuals with 3q29del reported significantly greater impairment than controls on several parent-report questionnaires that assessed different domains of social behavior, including social responsiveness and social communication [6]. This held true even in the absence of an ASD diagnosis in the proband. However, the core measure used in this study, the Social Responsiveness Scale (SRS), can show elevated T-scores in the presence of multiple neurodevelopmental and neuropsychiatric disorders, and may not be specific to ASD [23–25]. Therefore, it is unclear whether these increased scores are due to ASD-related symptoms specifically, or elevated social disability more generally. Further, this study relied on parent-report questionnaires, which can be biased [26]. Thus, the elevated social disability observed in individuals with 3q29del without an ASD diagnosis may be due to the presence of co-occurring neurodevelopmental or psychiatric conditions or may simply be driven by parental bias in reporting. Alternatively, the elevated social disability may be due to undiagnosed ASD within the 3q29del population.

Individuals with 3q29del and their families face significant challenges due to the neuropsychiatric and neurodevelopmental aspects of the syndrome, including significant social disability. These challenges impact multiple areas of day-to-day life, including school performance and the ability to function independently. It is well established that children with ASD experience more school-related problems, and adults with ASD and comorbid anxiety experience significantly worse quality of life [27–29]. However, the impact of social disability outside the context of frank ASD on quality of life is not well defined. Clinical observations by our team [17] found that individuals with 3q29del commonly have a lack of social awareness and inability to read social cues, while at the same time many individuals with 3q29del have the cognitive and adaptive ability to live semi-independently. This makes individuals with 3q29del uniquely vulnerable to manipulation and other social harms, particularly when an individual with 3q29del is engaging with the world independently of their caregivers, such as while traveling or at work. A previous study by our team found that caregivers of individuals with 3q29del experience a high degree of stress surrounding multiple aspects of the syndrome, including the burden of caring for a child with special needs and concerns about their child’s future ability to function independently [30]. These data suggest that an improved understanding of social disability in 3q29del, coupled with improved therapeutic strategies, may improve the quality of life for the affected individual and their family.

While studies utilizing parent-report questionnaires can provide broad insight into social disability phenotypes within the 3q29del population, a better understanding may be achieved via direct clinical evaluation. In the current study, we used gold-standard diagnostic instruments administered by our team of expert clinicians to explore nuances of social disability specifically relevant to ASD within our 3q29del study participants. Specifically, we sought to compare the social disability profile of individuals with 3q29del and a diagnosis of ASD to individuals with non-syndromic ASD, as assessed by the Autism Diagnostic Observation Schedule, Second Edition (ADOS-2), and the Autism Diagnostic Interview, Revised (ADI-R). We also compared the ADOS-2 performance from individuals with 3q29del who did not qualify for an ASD diagnosis to a set of individuals without 3q29del who do not have an ASD diagnosis. Developing a better understanding of the ASD phenotype in all individuals with 3q29del will help improve clinical care and quality of life for affected individuals. Further, our use of gold-standard evaluations will facilitate cross-disorder comparison to define core ASD phenotypes across genetic syndromes, as well as areas of phenotypic divergence ripe for future investigation.

## Methods

### Sample

Thirty-two individuals with 3q29del (Additional file 1: Table S1) were recruited through the online 3q29 deletion registry (3q29deletion.org) [5] for in-person evaluation at the Marcus Autism Center in Atlanta, GA [31]. One individual was evaluated using the ADOS-2 Module 1 and was excluded from the current analysis due to low item overlap with ADOS-2 Modules 2–4. Thirty-one individuals with 3q29del (61.3% male) were included in the present study, ranging in age from 4.9 to 39.1 years (mean = 14.59 ± 8.38 years). ASD diagnosis for individuals with 3q29del was reached via clinician’s best estimate, informed by the Autism Diagnostic Observation Schedule, Second Edition (ADOS-2) [32], and Autism Diagnostic Interview, Revised (ADI-R) [17, 33, 34]. Comparison samples of study subjects were ascertained from the National Database for Autism Research (NDAR), matched on age, sex, and ASD diagnosis status, and on race and ethnicity when possible (see Supplement for details). To maximize power, four comparison participants were selected for each 3q29del participant. The comparison sample for participants with 3q29del and a clinical ASD diagnosis (3q29del + ASD, *n* = 12) were individuals with a diagnosis of non-syndromic ASD (nsASD, *n* = 48); comparators for participants with 3q29del without a clinical ASD diagnosis (3q29del-ASD, *n* = 19) were typically developing (TD, *n* = 76) individuals. Individuals in NDAR with any reported neurodevelopmental concern or diagnosis were excluded from the TD group. A description of the study sample is given in Table 1. This study was approved by Emory University’s Institutional Review Board (IRB00064133) and Rutgers University’s Institutional Review Board (PRO2021001360).

**Table 1:** Participant demographics

	ADOS (N = 155)			ADI-R (N = 55)		
	3q29del (*n* = 31)	Comparator (*n* = 124)	*p* value	3q29del + ASD (*n* = 11)	Comparator (*n* = 44)	*p* value
Sex						
Male *n* (%)	19 (61.30)	19 (61.30)	1.00	9 (81.82)	36 (81.82)	1.00
Age						
Mean ± SD	14.59 ± 8.38	14.45 ± 8.32	0.94	14.75 ± 5.11	14.74 ± 5.00	1.00
Race						
White *n* (%)	28 (90.32)	104 (83.87)	1.00	9 (81.82)	36 (81.82)	1.00
Black *n* (%)	0 (0.00)	1 (0.81)		0 (0.00)	0 (0.00)	
Other *n* (%)	3 (9.68)	16 (12.90)		2 (18.18)	7 (15.91)	
Unknown/not reported *n* (%)	0 (0.00)	3 (2.42)		0 (0.00)	1 (2.27)	
Ethnicity						
Hispanic/Latino *n* (%)	1 (3.23)	3 (2.42)	1.88E-06	0 (0.00)	0 (0.00)	0.18
Not Hispanic/Latino *n* (%)	30 (96.77)	71 (57.26)		11 (100.00)	34 (77.27)	
Unknown/not reported *n* (%)	0 (0.00)	50 (40.32)		0 (0.00)	10 (22.73)	
Diagnosis						
ASD *n* (%)	12 (38.71)	48 (38.71)	1.00	11 (100.00)	44 (100.00)	1.00
IQ						
Mean ± SD	74.10 ± 13.05	103.09 ± 20.79	1.66E-14	69.82 ± 13.61	103.60 ± 16.48	1.35E-06

Demographic data collected through the online 3q29 registry (for 3q29del participants) or reported to NDAR (for comparator participants). *p* values were calculated with Fisher’s exact test (diagnosis, sex, race, and ethnicity) and two-sample t test (age). For race, “Other” includes Hawaiian or Pacific Islander (*n* = 1 comparator ADOS-2), more than one race (*n* = 3 3q29del ADOS-2, 11 comparator ADOS-2, 2 3q29del ADI-R, 7 comparator ADI-R), American Indian or Alaska Native (*n* = 2 comparator ADOS-2), and Asian (*n* = 2 comparator ADOS-2). For IQ, 15 ADOS-2 comparators (*n* = 9 nsASD and 6 TD) and 2 ADI-R comparators (*n* = 2 nsASD) did not have full-scale IQ or an equivalent reported in NDAR

### Measures

Gold-standard clinical evaluation of ASD symptomatology was performed using the Autism Diagnostic Observation Schedule, Second Edition [32] (ADOS-2, *n* = 12 3q29del + ASD, 19 3q29del-ASD, 48 nsASD, 76 TD), and Autism Diagnostic Interview, Revised [33, 34] (ADI-R, *n* = 11 3q29del + ASD, 44 nsASD). Additional detail regarding the administration and scoring of the ADOS-2 and ADI-R can be found in the Supplemental Methods.

#### ADOS-2

The ADOS-2 is a semi-structured, play-based, observational assessment of social interaction, communication, play and imagination skills, and repetitive behaviors. There are different modules for the ADOS-2 corresponding to the subject’s age and language level: Module 1 is used for nonverbal or minimally verbal individuals who are at least 31 months of age, Module 2 for individuals who speak in words and phrases of any age, Module 3 for individuals who are verbally fluent up to older adolescence, and Module 4 for individuals who are verbally fluent and are older adolescents or adults. Within each module, items are grouped into four categories: language and communication, reciprocal social interaction, play/imagination, and stereotyped behaviors and restricted interests. Higher domain and item scores correspond to greater impairment.

#### ADI-R

The ADI-R is a semi-structured interview between a parent and clinician focused on the early developmental history and current and lifetime behavior of the subject. The diagnostic algorithm of the ADI-R was used in the present study, which focuses on symptom presentation in early childhood. Items on the ADI-R are grouped into four domains for scoring: qualitative abnormalities in reciprocal social interaction; qualitative abnormalities in communication; restricted, repetitive, and stereotyped patterns of behavior; and abnormality of development evident at or before 36 months. The first three domains are further divided into sub-domains that capture different aspects of the domain. Higher domain, sub-domain, and item scores indicate greater symptom severity.

Despite comprehensive searches in NDAR, there were not enough TD subjects with ADI-R data, so a comparison of ADI-R performance between 3q29del cases without ASD and TD subjects was not made. ADI-R performance for 3q29del cases without ASD is summarized in Additional file 1: Table S4.

### Analysis

All analyses were performed in R version 4.0.4 [35]. For the ADOS-2, analyses were performed at the domain and item level. Domain-level Social Affect (SA) and Restricted and Repetitive Behaviors (RRB) raw scores were calculated according to the module-appropriate algorithms and were converted to Calibrated Severity Scores (CSS) for analysis across modules [32, 36, 37]. ADOS-2 item-level analyses were performed on a harmonized set of items with consistent names, descriptions, and scoring guidelines across ADOS-2 Modules 2–4 (Table 2). Exceptions for item matching were made if wording differences were due to the developmental nature of the module, but the item was capturing the same ability. Items from the “Other Abnormal Behaviors” section were excluded, as these items do not assess ASD-specific behaviors. Item-level harmonization (Table 2) was confirmed by trained clinicians, one of whom is a certified ADOS-2 Trainer (CK). For the ADI-R, domains were calculated according to the companion scoring algorithm [33, 34]. Sub-domain and item-level analyses were performed on any domains that showed a significant difference between 3q29del + ASD and nsASD participants.

**Table 2:** ADOS-2 item harmonization

ADOS-2 sections	Core item number	Item name			% Completed
		Module 2	Module 3	Module 4	
Language and Communication		Overall level of non-echoed spoken language	Overall level of non-echoed spoken language	Overall level of non-echoed spoken language	
	**1**	**Speech abnormalities associated with autism (intonation/volume/rhythm/rate)**	**Speech abnormalities associated with autism (intonation/volume/rhythm/rate)**	**Speech abnormalities associated with autism (intonation/volume/rhythm/rate)**	**92.90**
	**2**	**Immediate echolalia**	**Immediate echolalia**	**Immediate echolalia**	**98.06**
	**3**	**Stereotyped/idiosyncratic use of words or phrases**	**Stereotyped/idiosyncratic use of words or phrases**	**Stereotyped/idiosyncratic use of words or phrases**	**98.71**
	**4**	**Conversation**	**Conversation**	**Conversation**	**95.48**
		Pointing	Asks for information	Asks for information	
	**5**	**Descriptive, conventional, instrumental, or informational gestures**	**Descriptive, conventional, instrumental, or informational gestures**	**Descriptive, conventional, instrumental, or informational gestures**	**98.71**
			Offers information	Offers information	
			Reporting of events	Reporting of events	
				Emphatic or emotional gestures	
Reciprocal Social Interaction	**6**	**Unusual eye contact**	**Unusual eye contact**	**Unusual eye contact**	**98.06**
	**7**	**Facial expressions directed to others**	**Facial expressions directed to examiner**	**Facial expressions directed to examiner**	**94.84**
	**8**	**Shared enjoyment in interaction**	**Shared enjoyment in interaction**	**Shared enjoyment in interaction**	**97.42**
		Response to name	Language production and linked nonverbal communication	Language production and linked nonverbal communication	
		Showing	Insight into typical social situations and relationships	Insight into typical social situations and relationships	
		Spontaneous imitation of joint attention	Comments on others' emotions/empathy	Comments on others' emotions/empathy	
	**9**	**Quality of social overtures**	**Quality of social overtures**	**Quality of social overtures**	**98.06**
	**10**	**Amount of social overtures/maintenance of attention (examiner)**	**Amount of social overtures/maintenance of attention**	**Amount of social overtures/maintenance of attention**	**83.87**
		Amount of social overtures/maintenance of attention (parent/caregiver)		Communication of own affect	
	**11**	**Quality of social response**	**Quality of social response**	**Quality of social response**	**98.06**
	**12**	**Amount of reciprocal social communication**	**Amount of reciprocal social communication**	**Amount of reciprocal social communication**	**98.06**
	**13**	**Overall quality of rapport**	**Overall quality of rapport**	**Overall quality of rapport**	**95.48**
		Response to joint attention		Responsibility	
Play/Imagination	**14**	**Imagination/creativity**	**Imagination/creativity**	**Imagination/creativity**	**98.06**
		Functional play with objects			
Stereotyped Behaviors and Restricted Interests	**15**	**Unusual sensory interest in play material/person**	**Unusual sensory interest in play material/person**	**Unusual sensory interest in play material/person**	**98.71**
	**16**	**Hand and finger and other complex mannerisms**	**Hand and finger and other complex mannerisms**	**Hand and finger and other complex mannerisms**	**98.71**
	**17**	**Self-injurious behavior**	**Self-injurious behavior**	**Self-injurious behavior**	**98.06**
	**18**	**Unusually repetitive interests or stereotyped behaviors**	**Excessive interest in or references to unusual or highly specific topics or objects or repetitive behaviors**	**Excessive interest in or references to unusual or highly specific topics or objects or repetitive behaviors**	**95.48**
			Compulsions or rituals	Compulsions or rituals	

ADOS-2 Modules 2–4 item harmonization. Harmonized items are bolded. The percentage of study subjects that were scored on each harmonized item is noted

Statistical analysis of demographic variables was performed using Fisher’s exact test and two-sample *t* test implemented using the stats R package [35]. To compare ADOS-2 and ADI-R variables between 3q29del and comparator groups, generalized linear models, cumulative link models, and simple linear models were implemented using the stats [35] and ordinal [38] R packages. For ADOS-2 items without sufficient variation, Wilcoxon rank sum tests were implemented using the stats R package [35]. Data visualization was performed using the plotly R package [39].

## Results

### ADOS-2 reveals nuances of social disability in individuals with 3q29del and ASD

**Fig. 1:**
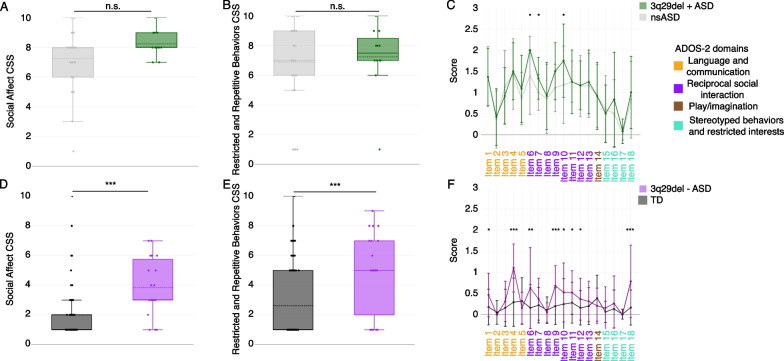
ADOS-2 domain and item scores. **A**, **B** Social affect (SA, **A**) and restricted and repetitive behaviors (RRB, **B**) calibrated severity scores (CSS) for individuals with 3q29del and ASD (*n* = 12) and nsASD comparators (*n* = 48), showing no significant difference in scores. **C** ADOS-2 harmonized item scores for individuals with 3q29del and ASD (*n* = 12) and nsASD comparators (*n* = 48), showing significantly increased scores in individuals with 3q29del and ASD on items 6, 7, and 10. **D**, **E** SA (**D**) and RRB (**E**) CSS for individuals with 3q29del and no ASD (*n* = 19) and TD comparators (*n* = 76), showing significantly increased scores in individuals with 3q29del and no ASD. **F** ADOS-2 harmonized item scores for individuals with 3q29del and no ASD (N = 19) and TD comparators (*n* = 76), showing significantly increased scores in individuals with 3q29del and no ASD on items 1, 4, 6, 9, 10, 11, 12, and 18. n.s., *p* > 0.05; *, *p* < 0.05; ***, *p* < 0.001

The ADOS-2 revealed no statistically significant differences between 3q29del participants with ASD and nsASD comparators on the SA (3q29del + ASD mean = 8.25 ± 0.87; nsASD mean = 7.26 ± 1.84; *p* > 0.05) or RRB (3q29del + ASD mean = 7.25 ± 2.26; nsASD mean = 6.89 ± 2.81; *p* > 0.05) domains (Fig. 1A,B). When comparing specific ADOS-2 items, 3q29del participants with ASD scored significantly higher (worse) than nsASD participants on items related to reciprocal social interaction: unusual eye contact (item 6, 3q29del + ASD mean = 2.00 ± 0.00; nsASD mean = 1.40 ± 0.92; *p* = 0.03), facial expressions directed toward others or the examiner (item 7, 3q29del + ASD mean = 1.33 ± 0.49; nsASD mean = 1.00 ± 0.43; *p* = 0.02), and amount of social overtures and maintenance of attention during the assessment (item 10, 3q29del + ASD mean = 1.75 ± 0.87; nsASD mean = 1.18 ± 0.91; *p* = 0.04) (Fig. 1C, Additional file 1: Figure S2). These data suggest that reciprocal social interaction may be a specific area of severe vulnerability for individuals with 3q29del and ASD.

### ADOS-2 reveals increased social disability in individuals with 3q29del and no ASD

Within the non-ASD group, 3q29del study subjects without an ASD diagnosis scored significantly higher than TD comparators on the SA domain (3q29del-ASD mean = 3.84 ± 1.95; TD mean = 2.01 ± 1.77; *p* = 4.24E-6), but not the RRB domain (3q29del-ASD mean = 5.00 ± 2.75; TD mean = 2.62 ± 2.46; *p* = 0.189) (Fig. 1D,E). At the item-level of analysis, 3q29del participants without ASD scored significantly higher than TD participants on 8 items: speech abnormalities associated with ASD (item 1, 3q29del-ASD mean = 0.47 ± 0.52; TD mean = 0.18 ± 0.42; *p* = 0.01), conversation (item 4, 3q29del-ASD mean = 1.11 ± 0.57; TD mean = 0.30 ± 0.57; *p* = 4.22E-7), unusual eye contact (item 6, 3q29del-ASD mean = 0.63 ± 0.96; TD mean = 0.16 ± 0.55; *p* = 0.007), the quality of social overtures (item 9, 3q29del-ASD mean = 0.68 ± 0.48; TD mean = 0.20 ± 0.44; *p* = 0.0001), the amount of social overtures and maintenance of attention (item 10, 3q29del-ASD mean = 0.53 ± 0.70; TD mean = 0.25 ± 0.60; *p* = 0.03), the quality of social response (item 11, 3q29del-ASD mean = 0.53 ± 0.51; TD mean = 0.28 ± 0.48; *p* = 0.04), the amount of reciprocal social communication (item 12, 3q29del-ASD mean = 0.37 ± 0.50; TD mean = 0.16 ± 0.41; *p* = 0.04), and repetitive interests and repetitive or stereotyped behaviors evident during the evaluation (item 18, 3q29del-ASD mean = 0.79 ± 0.85; TD mean = 0.17 ± 0.41; *p* = 0.0003) (Fig. 1F, Additional file 1: Figure S3). These data indicate that for individuals with 3q29del, significant social disability may be present even in the absence of an ASD diagnosis.

### ADI-R shows 3q29del participants with ASD perform better on social communication than nsASD

**Fig. 2:**
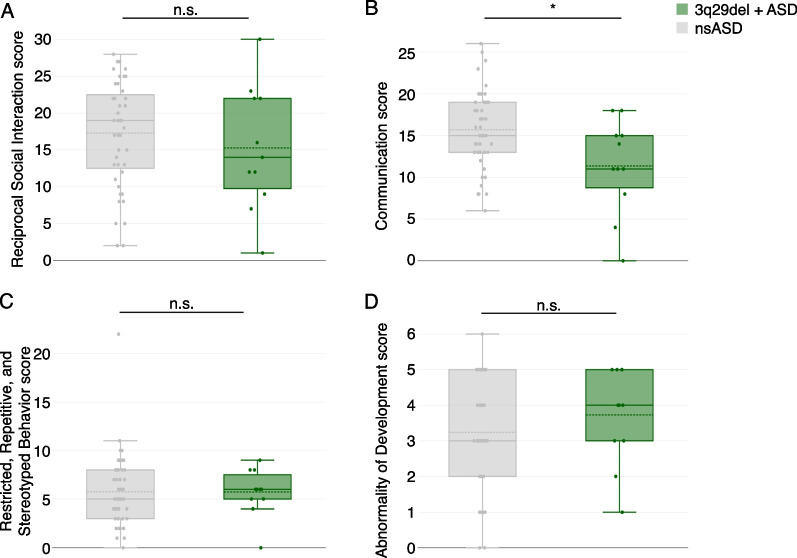
ADI-R domain scores. **A** ADI-R domain A scores, showing no significant difference between individuals with 3q29del and ASD (*n* = 11) and nsASD comparators (*n* = 44). **B** ADI-R domain B scores, showing significantly lower scores in individuals with 3q29del and ASD (*n* = 11) compared to nsASD comparators (*n* = 44). **C** ADI-R domain C scores, showing no significant difference between individuals with 3q29del and ASD (*n* = 11) and nsASD comparators (*n* = 44). **D** ADI-R domain D scores, showing no significant difference between individuals with 3q29del and ASD (*n* = 11) and nsASD comparators (*n* = 44). n.s., *p* > 0.05; *, *p* < 0.05

To further define nuances of the social disability phenotype within our population of individuals with 3q29del and ASD, we examined data from the ADI-R. Of the four core domains assessed by the ADI-R, only one domain was significantly different between 3q29del participants with ASD and nsASD participants (Fig. 2). 3q29del participants with ASD scored significantly lower (better) than nsASD participants on domain B, corresponding to “qualitative abnormalities in communication” (3q29 + ASD mean = 11.36 ± 5.63; nsASD mean = 15.70 ± 4.75; *p* = 0.01; Fig. 2B), indicating less impairment on average in social communication in individuals with 3q29del and ASD compared to nsASD subjects.

**Fig. 3:**
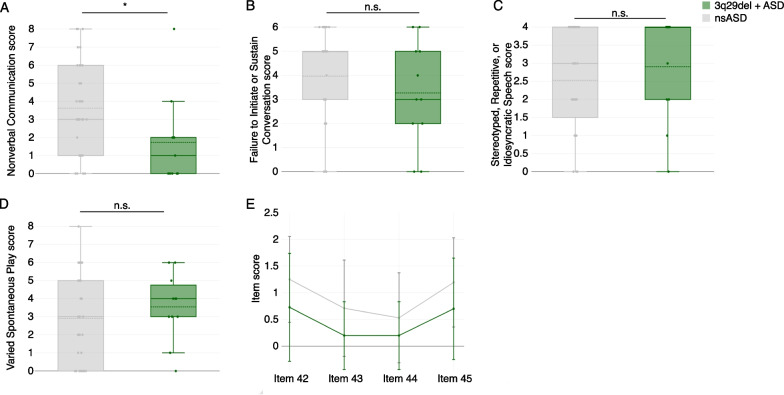
ADI-R sub-domain and item scores. **A** ADI-R sub-domain B1 scores, showing significantly lower scores in individuals with 3q29del and ASD (*n* = 11) compared to nsASD comparators (*n* = 32). **B** ADI-R sub-domain B2 scores, showing no significant difference between individuals with 3q29del and ASD (*n* = 11) and nsASD comparators (*n* = 32). **C** ADI-R sub-domain B3 scores, showing no significant difference between individuals with 3q29del and ASD (*n* = 11) and nsASD comparators (*n* = 32). **D** ADI-R sub-domain B4 scores, showing no significant difference between individuals with 3q29del and ASD (*n* = 11) and nsASD comparators (*n* = 32). **E** ADI-R sub-domain B1 item scores for individuals with 3q29del and ASD (*n* = 11) and nsASD comparators (*n* = 32), showing no significant difference on any sub-domain items. n.s., *p* > 0.05; *, *p* < 0.05

At the domain level, it was unclear whether the significantly reduced domain B score in individuals with 3q29del and ASD was due to decreased scores across multiple factors, or a specific difference in a single sub-domain. Analysis of the four sub-domains that comprise domain B revealed that only one was significantly lower in participants with 3q29del and ASD compared to nsASD (Fig. 3A–D). 3q29del participants with ASD scored significantly lower than nsASD participants on sub-domain B1, “lack of, or delay in, spoken language and failure to compensate through gesture” (3q29 + ASD mean = 1.73 ± 2.45; nsASD mean = 3.63 ± 2.54; *p* = 0.02; Fig. 3A). Sub-domain B1 is comprised of four items: “pointing to express interest” (item 42), “nodding” (item 43), “head shaking” (item 44), and “conventional/instrumental gestures” (item 45). Within sub-domain B1, participants with 3q29del and ASD scored slightly lower on average than nsASD participants on all four items, but the differences were not statistically significant (Fig. 3E).

### Speech delay is not related to higher nonverbal communication scores in 3q29del

**Fig. 4:**
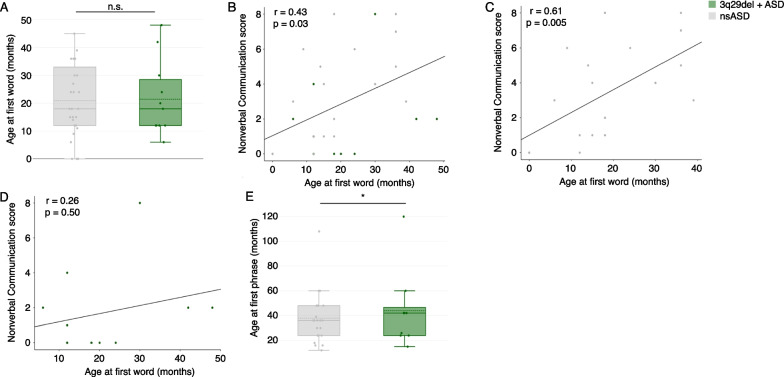
Speech delay and ADI-R nonverbal communication scores. **A** Age at first single word (months) is not significantly different between individuals with 3q29del and ASD (*n* = 11) and nsASD comparators (*n* = 32). **B** Relationship between age at first single word (months) and sub-domain B1 scores in the full dataset (3q29del + ASD *n* = 11; nsASD *n* = 32), showing a significant positive relationship. **C** Relationship between age at first single word (months) and sub-domain B1 scores in nsASD comparators (*n* = 32), showing a significant positive relationship. **D** Relationship between age at first single word (months) and sub-domain B1 scores in individuals with 3q29del and ASD (*n* = 11), showing no significant relationship. **E** Age at first two-word phrase (months) is significantly later in individuals with 3q29del and ASD (*n* = 11) compared to nsASD comparators (*n* = 32). n.s., *p* > 0.05; *, *p* < 0.05

We hypothesized that the use of nonverbal communication strategies may be related to the degree of speech delay in our study participants. Specifically, we asked whether individuals with a more significant speech delay compensated for their lack of speech through the use of stronger nonverbal communication skills. To investigate this hypothesis, we analyzed the age of first single word and the age of first two-word phrases as recorded on the ADI-R, in relation to ADI-R domain B (social communication) and sub-domain B1 (nonverbal communication) scores. We found that there was no genotype difference in age at first single word (3q29del + ASD mean = 21.45 ± 13.48 months; nsASD mean = 20.93 ± 12.75 months; *p* = 0.88; Fig. 4A). After adjusting for genotype, age at first single word was significantly associated with the nonverbal communication score (*p* = 0.03; Fig. 4B, Additional file 1: Table S5). In genotype-specific analyses, age at first single word was significantly associated with nonverbal communication scores in nsASD comparators (*p* = 0.005; Fig. 4C, Additional file 1: Table S5), but the association was not statistically significant in individuals with 3q29del and ASD (Fig. 4D, Additional file 1: Table S5). On average, individuals with 3q29del and ASD spoke their first two-word phrase approximately 6 months later than nsASD comparators (3q29del + ASD mean = 43.89 ± 31.64 months; nsASD mean = 37.86 ± 20.76 months; *p* = 0.01; Fig. 4E). However, age at first two-word phrase was not significantly associated with social communication or nonverbal communication scores (Additional file 1: Table S5). The lack of association between age at first two-word phrase and the social communication and nonverbal communication scores demonstrates that speech delay cannot account for the less-impacted nonverbal communication in our 3q29del participants.

## Discussion

The present study is the first to use gold-standard ASD evaluations to assess features of social disability in individuals with 3q29del. Individuals with 3q29del and ASD are largely indistinguishable from individuals with nsASD on both the ADOS-2 (Fig. 1A–C) and ADI-R (Figs. 2 and 3), with some minor differences on specific items. In contrast, individuals with 3q29del and no ASD had substantially more social disability than TD comparators on the ADOS-2, with scores for the SA and RRB domains as well as significantly elevated scores on nearly 50% of the 18 harmonized ADOS-2 items (Fig. 1D–F). These findings are consistent with previous work by our group, in which we found significant social disability within our 3q29del patient population independent of ASD diagnosis relative to TD controls [6].

There were some specific areas of divergence between individuals with 3q29del and ASD and nsASD comparators. On the ADOS-2, individuals with 3q29del and ASD scored significantly higher than nsASD comparators on 3 harmonized items related to social reciprocity during the evaluation, namely unusual eye contact, facial expressions directed toward the examiner, and the frequency of social overtures and maintenance of attention during the assessment. These data suggest that individuals with 3q29del and ASD are uniquely impaired in these areas, with greater impairment on average than is observed in children with nsASD. Conversely, on the ADI-R, individuals with 3q29del and ASD scored significantly lower than nsASD comparators on the social communication domain, specifically on nonverbal communication, indicating that these domains are relatively well preserved in individuals with 3q29del and ASD relative to individuals with nsASD. This seeming contradiction between the ADOS-2 and ADI-R results may be due to several possible factors. First, the ADOS-2 is a direct clinical assessment of the affected individual, whereas the ADI-R is a historical parent-report interview. Second, our team has observed that many individuals with 3q29del are well bonded to their caregivers; it is possible that social disability may be more significant in new social interactions. A previous analysis of the Social Communication Questionnaire (SCQ) by our team revealed that our 3q29del study population as a whole had mean scores in the normal range, and individuals with 3q29del and ASD had scores only slightly above the clinical cutoff [6]. Additionally, clinical evaluations by our team found that verbal IQ in 3q29del is significantly higher than nonverbal IQ [16]. Together with our current finding that nonverbal social communication is relatively less impaired within our sample of individuals with 3q29del and ASD, these data suggest that social disability within the 3q29del population is not due to deficits in frank verbal ability.

The high prevalence of social disability in individuals with 3q29del and no ASD diagnosis is a critical consideration in this population. Typically, a diagnosis of ASD is required to qualify for social skills interventions and other therapeutic services. However, a majority of individuals with 3q29del do not qualify for an ASD diagnosis, even in the presence of a substantial social disability. Previous work by our team [6] in combination with the present work highlights the critical need for early, gold-standard ASD evaluation for individuals diagnosed with 3q29del. The ADOS-2 and ADI-R are the gold-standard instruments for assessing autism symptomatology in ASD and other neurodevelopmental disorders and have been validated across many genetic disorders that are strongly associated with ASD [40–43]. Further, the high degree of social disability independent of ASD diagnosis demonstrates that a diagnosis of 3q29del alone should be sufficient for an individual to qualify for social skills interventions and other early intervention strategies. Alternatively, the Diagnostic and Statistical Manual 5 (DSM-5) diagnosis “Unspecified Neurodevelopmental Disorder” may be considered in a clinical setting for an individual with 3q29del that does not fully reach the diagnostic criteria for ASD. The high prevalence of social disability associated with 3q29del also suggests that the 3q29 deletion impacts key biological pathways relevant to social function. Thus, the 3q29 deletion may serve as a window to understanding the molecular pathology underlying social disability.

There has been significant research effort into social skills interventions for school-age students with ASD [28, 44–47]. The high prevalence of social disability associated with 3q29del suggests that interventions primarily designed for children with ASD may be universally beneficial for this group. While interventions are typically studied and implemented in an academic setting, the basic tenants of these strategies can also be applied in other environments. Frameworks such as “tell, show, do, follow through and practice, and generalization” [44] could be beneficial for individuals with 3q29del both at home and at school, as a way to help the individual practice appropriate social interaction. Increased education surrounding the social skills needs of individuals with 3q29del for educators and clinicians will be central to the successful implementation of these strategies. Critically, to avoid unnecessary caregiver burden, these interventions should be applied across a variety of contexts (educational, therapeutic, and home), rather than relying on the caregiver to be the sole provider of social skills training.

Previous work by our group using parent-report questionnaires identified a high burden of social disability within our 3q29del study population; however, average scores for social communication were within the normal range [6]. The present study supports this finding and further suggests that social communication is better preserved in individuals with 3q29del and ASD than other aspects of social behavior, and nonverbal communication ability appears to drive this preservation. We hypothesized that early speech delay in our 3q29del participants may incentivize the development of nonverbal communication skills as a compensatory mechanism. Study subjects with 3q29del and ASD had significantly later age at first two-word phrase compared to nsASD comparators (Fig. 4E); however, this delay was not associated with measures of nonverbal communication in our 3q29del participants. Based on these data, speech delay alone is not sufficient to explain the relatively preserved nonverbal communication in individuals with 3q29del and ASD compared to nsASD comparators. Further, because nonverbal social communication is relatively well preserved in individuals with 3q29del, other ASD symptoms may be overlooked. This highlights the critical need for gold-standard ASD evaluations as a standard of care for all individuals diagnosed with 3q29del.

The results of the present study show that the social disability associated with 3q29del is qualitatively distinct from that observed in several other rare genetic disorders. A study of individuals with tuberous sclerosis complex found that the ASD symptom profile was largely concordant with nsASD comparators, especially regarding social communication [42]. Individuals with Phelan-McDermid syndrome typically show the greatest impairment in social communication and nonverbal communication [41, 48]. While Williams syndrome is canonically associated with inappropriate social behavior and hyper-sociability [49–51], a study of young children with Williams syndrome identified specific difficulties in nonverbal social communication [52]. 22q11.2 duplication syndrome has an ASD symptom profile more similar to 3q29del, with social communication relatively well preserved relative to other ASD symptom domains [53]. However, while these qualitative differences exist between the syndromes, it is important to note they are all associated with significant social disability [41, 42, 48–53], highlighting the value of rare diseases as a model of social disability. To maximize the translational impact of future studies of rare genetic disorders, several research frameworks have been developed to help guide study design and facilitate cross-disorder comparison [54, 55]. Rapidly expanding research efforts in this area will help to improve our current understanding of social disability and ASD phenotypes, and ideally will help to shape more targeted therapeutic strategies for affected individuals.

Taken together, the data presented in the current study reveal substantial social disability within the 3q29del population as assessed by gold-standard ASD evaluations. This finding is supported by a previous study by our team that examined parent-report questionnaires that focused on social disability phenotypes [6]. Coupled with our previous work, the present findings emphasize the need for early gold-standard ASD evaluation for all individuals with a diagnosis of 3q29 deletion syndrome. Additionally, these data support the need for social skills interventions within the 3q29del population, even in the absence of a clinical ASD diagnosis. Individuals with 3q29del and no ASD diagnosis may be uniquely vulnerable to social disability because individuals without an ASD diagnosis are traditionally not prioritized for social skills interventions. By prioritizing early social skills therapies for all individuals with 3q29del, we may improve the social function of the entire population of individuals with 3q29del, rather than only focusing on those that qualify for a clinical ASD diagnosis.

## Limitations

While this study contributes valuable information to our understanding of social disability within the 3q29del population, it is not without limitations. There were some challenges with identifying comparators in NDAR. First, we were unable to identify a sufficient number of TD comparators with an ADI-R within the database, which caused us to restrict our ADI-R analysis to only individuals with 3q29del and ASD and nsASD comparators. Second, the TD comparators that were identified for the ADOS-2 analyses may not truly be TD. While no ASD or other clinical diagnosis was noted, it is unclear whether some of these individuals received an ADOS-2 evaluation because of other developmental concerns that did not meet the threshold for diagnosis. However, even if there is some developmental concern within the TD group, individuals with 3q29del and no ASD diagnosis still showed significantly more impairment on the ADOS-2. This suggests that this limitation may not have substantially impacted the core findings of the present study. Third, we were unable to identify comparators that were matched on IQ. The comparator groups in our analysis had significantly higher IQ as compared to our 3q29del study participants (Table 1). However, when we tested all of the models in our analysis, we found that IQ only significantly improved the model fit for one item (Additional file 1: Table S6) and including IQ in that analysis did not change the interpretation of the data. This implies that while IQ was different between the 3q29del and non-3q29del groups, it did not meaningfully impact the results of our analysis. Finally, we were unable to assess race and ethnicity effects in the present study, due to the small sample size and a lack of diversity within the 3q29del registry. Future effort is required to reach a more diverse population of individuals with 3q29del. Additionally, systemic changes are required to address long-standing disparities in the access to and utilization of genetic services, which currently may contribute to under-diagnosis of genetic and genomic syndromes like 3q29del within minority populations [56–60].

## Conclusions

The present study is the first to examine nuances of social disability within the 3q29del population using gold-standard ASD evaluations. We also present a harmonized approach for analyzing item-level data across ADOS-2 modules 2, 3, and 4. We find significant social disability present in our 3q29del study population; individuals with 3q29del and ASD have a similar degree of social disability to nsASD comparators, while individuals with 3q29del and no ASD have a substantially greater social disability than TD comparators. Additionally, individuals with 3q29del and ASD have relatively well-preserved social communication, specifically nonverbal communication, and relatively impaired social reciprocity, compared to nsASD comparators. We hypothesize that ASD and social disability may be under-appreciated in some cases of 3q29del due to the relatively preserved verbal ability and social communication within this population. This hypothesis is supported by anecdotal reports from parents of children with 3q29del, who recount having their concerns about ASD in their child dismissed because their child was considered “too verbal” by their clinician. As verbal ability is not a diagnostic criterion for ASD, this clinical interpretation is inappropriate and highlights the need for improved clinical education surrounding the presentation of social disability in 3q29del. Based on these data and previous work by our team [6, 17], we recommend that all individuals with 3q29del should be referred to experts to receive gold-standard ASD evaluations as a standard of care, regardless of the individual’s verbal ability. Early diagnosis of social deficits and early therapeutic intervention within this patient population will be the most effective way to improve future outcomes and quality of life for individuals with 3q29del and their families.

## Supplementary Information

**Additional file 1**. Supplemental methods, figures S1-S3, and tables S1-S6.

## Data Availability

The datasets used and/or analyzed during the current study are available from the corresponding author upon reasonable request.

## Abbreviations

3q29del: 3q29 deletion syndrome
ASD: Autism spectrum disorder
SZ: Schizophrenia
ID: Intellectual disability
GDD: Global developmental delay
TD: Typically developing
SRS: Social Responsiveness Scale
ADOS-2: Autism Diagnostic Observation Schedule, Second Edition
ADI-R: Autism Diagnostic Interview, Revised
NDAR: National Database for Autism Research
nsASD: Non-syndromic autism spectrum disorder
SA: Social affect
RRB: Restricted and repetitive behaviors
CSS: Calibrated severity score
SCQ: Social Communication Questionnaire
