# Supplementary material for: Autism spectrum disorder symptom expression in individuals with 3q29 deletion syndrome
Source: Mol Autism. 2022 Dec 24;13:50. doi: 10.1186/s13229-022-00533-2 (PMC9789637; doi:10.1186/s13229-022-00533-2)
Supplement: Supplementary file 1 — Additional file 1. Supplemental methods, figures S1-S3, and tables S1-S6. [file 13229_2022_533_MOESM1_ESM.docx]

**Supplemental Methods**

*Matching comparators for 3q29del participants*

Four comparators for each 3q29del participant were ascertained from the National Database for Autism Research (NDAR, Table S2). Comparators for participants with 3q29del and a clinical ASD diagnosis (3q29del+ASD, n = 12) were individuals with non-syndromic ASD (nsASD); comparators for participants with 3q29del without a clinical ASD diagnosis (3q29del-ASD, n=19) were TD individuals. All comparators were matched on age, sex, and ASD diagnosis status, and were matched on race and ethnicity when possible. For matching on age, individuals under 18 were matched on exact age, meaning a match for an 8 year old individual would be between 8 years, 0 months, and 8 years, 11 months. Individuals over 18 were matched within a two year range, centered on their age, meaning a match for a 24 year old individual would be between 23 years, 0 months, and 25 years, 11 months. Exceptions for age matching were made for 7 participants in the ADOS-2 data set (Table S3). One participant with 3q29del (a 34 year old female) was excluded from the ADI-R data set due to a lack of reasonable age-matched comparators in NDAR. No exceptions for age matching were made in the final ADI-R data set.

When possible, the same comparators were used in the ADOS-2 and ADI-R datasets. However, there was limited overlap between the comparator data sets due to differences in data availability for studies in NDAR (Figure S1).

*ADOS-2*

The ADOS-2 is a semi-structured, standardized, observational assessment of social interaction, communication, play and imagination skills, and repetitive behaviors. During the assessment, the subject is placed in different play-based situations. The clinician does not provide the subject with any instructions or guidance in order to observe the subject’s natural behaviors. There are different modules for the ADOS-2 corresponding to the subject’s age and language level: Module 1 is used for nonverbal or minimally verbal individuals who are at least 31 months of age, Module 2 for individuals who speak in words and phrases of any age, Module 3 for individuals who are verbally fluent up to older adolescence, and Module 4 for individuals who are verbally fluent and are older adolescents or adults. Within each module, items are grouped into four categories: language and communication, reciprocal social interaction, play/imagination, and stereotyped behaviors and restricted interests. A subset of the scored items are used to calculate two domain scores, according to a standard algorithm (1). The domains are Social Affect (SA) and Restricted and Repetitive Behavior (RRB). Higher domain and item scores correspond to greater impairment.

For analysis, SA and RRB domain scores were calculated according to the companion scoring algorithms (1, 2). Because the items used to calculate the SA and RRB domain scores vary between modules, the domain scores were converted to Calibrated Severity Scores (CSS) for comparison across modules (1-3). Possible CSS values range from 0 to 10. Item-level analyses were performed on core items across modules (Table 2). Possible item scores range from 0 to 3. For item-level analysis, scores between 0 and 3 were preserved and scores of 7, 8, or 9 were dropped.

*ADI-R*

The ADI-R is a semi-structured interview between a parent and clinician focused on the early developmental history and current and lifetime behavior of the subject. The diagnostic algorithm of the ADI-R was used in the present study, which focuses on symptom presentation in early childhood. Items on the ADI-R are grouped into four domains for scoring: qualitative abnormalities in reciprocal social interaction; qualitative abnormalities in communication; restricted, repetitive, and stereotyped patterns of behavior; and abnormality of development evident at or before 36 months. The first three domains are further divided into sub-domains that capture different aspects of the domain. Scores are calculated according to the companion scoring algorithm (4, 5). Higher domain, sub-domain, and item scores indicate greater symptom severity.

For analysis, domain scores were calculated according to the companion scoring algorithm (4, 5). Analysis was performed at the domain level first; any domains showing a significant difference between individuals with 3q29del and ASD and nsASD comparators were broken into sub-domains. If any sub-domains showed a significant difference, the individual items of that sub-domain were examined. The score range for domains and sub-domains varies according to the number of items included in each domain. Item-level scores range from 0 to 3. For item-level analysis, scores between 0 and 3 were preserved and scores of 7, 8, or 9 were dropped.

**Table S1. Genetic testing results for study participants with 3q29del.**

| Sex | Age at genetic testing (years) | Coordinates | Deletion size (Mb) | Genetic testing method |
| --- | --- | --- | --- | --- |
| Male | 12 | hg38, chr3:196013531-197590232 | 1.58 | Microarray |
| Male | 2 | hg38, chr3:196035452-197620100 | 1.60 | SNP array |
| Male | 10 | hg38, chr3:196042699-197606105 | 1.56 | Microarray |
| Male | 2 | hg38, chr3:195963370-197572940 | 1.60 | Microarray |
| Female | 13 | hg38, chr3:195963356-197629463 | 1.67 | Microarray |
| Male | 2 | hg38, chr3:195997494-197646984 | 1.65 | Microarray |
| Female* | 15 | ish del(3)(q29q29)(RP11-962B7-)dn | 1.63 | Microarray, confirmed by FISH |
| Female | 13 | hg38, chr3:196044872-197358752 | 1.30 | Array CGH |
| Male | 12 | hg38, chr3:195976744-197617305 | 1.60 | SNP array |
| Male | 4 | hg38, chr3:195974280-197627105 | 1.65 | Microarray |
| Male | 10 | hg38, chr3:196053409-197572881 | 1.50 | Microarray |
| Male | 8 | hg38, chr3:195939241-197667295 | 1.73 | Array CGH |
| Male | 2 | hg38, chr3:195973827-197659309 | 1.69 | Microarray |
| Female | 6 | hg38, chr3:195984448-197627104 | 1.64 | Microarray |
| Female | 1 | hg38, chr3:196013486-197590232 | 1.58 | Array CGH |
| Female | 3 | hg38, chr3:195976744-197621704 | 1.65 | Microarray |
| Male | 1 | hg38, chr3:196013531-197590232 | 1.58 | Array CGH |
| Male | 16 | hg38, chr3:196042699-197606105 | 1.56 | Array CGH |
| Female | 3 | hg38, chr3:195991874-197629463 | 1.60 | Microarray |
| Female | 27 | hg38, chr3:195992934-197637631 | 1.70 | Microarray |
| Male | 7 | hg38, chr3:195973827-197621690 | 1.65 | Microarray |
| Female | 19 | hg38, chr3:196008062-197627105 | 1.62 | Microarray |
| Male | 2 | hg38, chr3:195939241-197667295 | 1.73 | FISH |
| Male | 15 | hg38, chr3:195998261-197629463 | 1.63 | Microarray |
| Female | 6 | hg38, chr3:195963101-197631263 | 1.66 | Microarray |
| Male | 11 months | hg38, chr3:196077857-197289753 | 1.20 | FISH |
| Male | 9 | hg38, chr3:195976744-197627104 | 1.65 | Microarray |
| Male | 3 | hg38, chr3:195998740-197667295 | 1.67 | Microarray |
| Female | 4 | hg38, chr3:195998740-197667295 | 1.67 | Microarray |
| Male | Birth | hg38, chr3:195998740-197667295 | 1.67 | Microarray |
| Male | 34 | hg38, chr3:195998740-197667295 | 1.67 | Microarray |
| Female | 9 | hg38, chr3:196013486-197590232 | 1.58 | Microarray |

*Exact genomic coordinates were unavailable for this participant; her medical record states she has a canonical 3q29 deletion. The confirmatory FISH probe is located inside the canonical interval.

**Table S2. NDAR data collections used to construct comparator datasets.**

| NDAR collection ID | NDAR collection title | ADOS-2 records (n) | ADI-R records (n) |
| --- | --- | --- | --- |
| 1 | UIC ACE: Translational Studies of Insistence on Sameness in Autism | 2 | 2 |
| 1889 | ASD Mathematical Cognition: A Cognitive and Systems Neuroscience Approach | 0 | 5 |
| 1985 | Evaluating the Time-Dependent Unfolding of Social Interactions in Children with Autism | 2 | 0 |
| 2001 | Influence of Attention and Arousal on Sensory Abnormalities in ASD | 0 | 2 |
| 2013 | Magnetoencephalographic studies of lexical processing and abstraction in autism | 1 | 0 |
| 2021 | Multimodal Developmental Neurogenetics of Females with ASD | 13 | 16 |
| 2025 | Minimally Verbal ASD: From Basic Mechanisms to Innovative Interventions | 1 | 1 |
| 2026 | Biomarkers of Developmental Trajectories and Treatment in ASD | 0 | 2 |
| 2030 | Electrophysiological Response to Executive Control Training in Autism | 1 | 4 |
| 2053 | Autism Spectrum Disorder: Birth Cohort 1976-2000, Epidemiology and Adult | 17 | 0 |
| 2066 | The CHARGE Study: Childhood Autism Risks From Genetics and the Environment | 3 | 0 |
| 2093 | Sporadic Mutations and Autism Spectrum Disorders | 4 | 0 |
| 2116 | Improving Transition Outcomes in ASD using COMPASS | 1 | 0 |
| 2120 | Adapting a Parent Advocacy Program to Improve Transition for Youth With Autism | 3 | 0 |
| 2179 | Neural markers of shared gaze during simulated social interactions in ASD \-Modal Automated Assessment of Behavior during Social Interactions in Children with ASD | 0 | 2 |
| 2266 | Inhibitory dysfunction in autism | 0 | 2 |
| 2282 | Cognitive Enhancement Therapy for Adult Autism Spectrum Disorder | 12 | 4 |
| 2285 | Integrity and Dynamic Processing Efficiency of Networks in ASD | 1 | 1 |
| 2288 | The Autism Biomarkers Consortium for Clinical Trials | 11 | 0 |
| 2292 | Molecular Mechanisms of Atypical Habituation in Autism Spectrum Disorders | 9 | 0 |
| 2293 | Components of Emotional Processing in Toddlers with ASD | 1 | 0 |
| 2368 | Clinical and Immunological Investigations of Subtypes of Autism | 13 | 0 |
| 2421 | Optimizing Prediction of Social Deficits in Autism Spectrum Disorders | 6 | 0 |
| 2441 | Structural and Functional Characteristics of XYY - Relationship to ASD | 1 | 0 |
| 2471 | A Simultaneous PET-MR Study of Striatal Dopamine Binding in Autism | 1 | 0 |
| 2706 | Thalamic activity and structure and surface neural oscillations in autism | 4 | 0 |
| 2711 | Motor abnormalities and functional brain mechanisms in autism spectrum disorder | 1 | 2 |
| 2761 | Charting the trajectory of executive control in autism in order to optimize delivery of intervention | 5 | 0 |
| 2771 | Emergent Gaze Perception in Autism Spectrum Disorder | 2 | 0 |
| 2778 | Heterogeneity in Autism Spectrum Disorders: Biological Mechanisms, Trajectories, and Treatment Response | 1 | 1 |
| 2821 | Cellular, molecular, and functional imaging approaches to understanding early neurodevelopment in autism | 5 | 0 |
| 2866 | Investigating Social Competence in Youth with Autism: A Multisite RCT | 1 | 0 |
| 2928 | Understanding Attentional Strengths and Weaknesses in Autism Spectrum Disorder | 2 | 0 |

List of NDAR data collections used to construct ADOS-2 and ADI-R comparator datasets.

**Table S3. Age-matching exceptions for ADOS-2 comparators.**

| 3q29del proband age (years) | Matched comparator exception | Group |
| --- | --- | --- |
| 4 | 1 month younger than criteria (3 years 11 months) | 3q29del-ASD |
| 6 | 19 months younger than criteris (4 years 5 months) | 3q29del-ASD |
| 6 | 15 months younger than criteria (4 years 9 months) | 3q29del-ASD |
| 15 | 2 months younger than criteria (14 years 10 months) | 3q29del-ASD |
| 14 | 5 months younger than criteria (13 years 7 months); 1 month older than criteria (15 years 0 months) | 3q29del-ASD |
| 34 | 4 months younger than criteris (32 years 8 months) | 3q29del+ASD |
| 39 | 3 months younger than criteria (37 years 9 months) | 3q29del-ASD |

Exceptions to age matching for comparators in ADOS-2 data set.

**Table S4. ADI-R performance for 3q29del cases without ASD.**

| Domain | | Score (mean ± SD) |
| --- | --- | --- |
| A (Reciprocal social interaction) | | 7.11 ± 6.93 |
| B (Communication) | | 5.06 ± 5.91 |
|  | B1 (Nonverbal communication) | 0.83 ± 1.98 |
|  | B2 (Failure to initiate or sustain conversation) | 1.67 ± 1.24 |
|  | B3 (Stereotyped, repetitive, or idiosyncratic speech) | 1.56 ± 2.01 |
|  | B4 (Varied spontaneous play) | 1.00 ± 1.81 |
| C (Restricted, repetitive, and stereotyped behavior) | | 3.06 ± 2.21 |
| D (Abnormality of development) | | 3.06 ± 0.94 |

Summary of ADI-R domain-level performance for study subjects with 3q29del and no ASD.

**Table S5. First single word and first two-word phrase model results.**

| Comparison | ADI-R Communication domain | | ADI-R Nonverbal Communication sub-domain | |
| --- | --- | --- | --- | --- |
|  | Estimate | P value | Estimate | P value |
| First single word, full data set | 0.051 | 0.48 | 0.076 | 0.03 |
| First single word, 3q29del+ASD only | -0.159 | 0.45 | -0.047 | 0.50 |
| First single word, nsASD only | 0.141 | 0.08 | 0.157 | 0.005 |
| First two-word phrase, full data set | 0.069 | 0.08 | 0.038 | 0.06 |
| First two-word phrase, 3q29del+ASD only | 0.098 | 0.32 | 0.017 | 0.47 |
| First two-word phrase, nsASD only | 0.066 | 0.22 | 0.110 | 0.01 |

Summary of analyses of the relationship age at first single word and age at first two-word phrase with ADI-R domain B and ADI-R sub-domain B1.

**Table S6. Goodness of fit analysis.**

| Analysis group | Full model | P value, age | P value, IQ | Final model |
| --- | --- | --- | --- | --- |
| ADOS-2, 3q29del with ASD vs. nsASD | SA CSS ~ Group + Module + Sex + Age + IQ | 0.969 | 0.345 | SA CSS ~ Group + Module + Sex |
|  | RRB CSS ~ Group + Module + Sex + Age + IQ | 0.165 | 0.152 | RRB CSS ~ Group + Module + Sex |
|  | Item 1 ~ Group + Module + Sex + Age + IQ | 0.390 | 0.388 | Item 1 ~ Group + Module + Sex |
|  | Item 2 ~ Group + Module + Sex + Age + IQ | NA | NA | NA |
|  | Item 3 ~ Group + Module + Sex + Age + IQ | 0.155 | 0.901 | Item 3 ~ Group + Module + Sex |
|  | Item 4 ~ Group + Module + Sex + Age + IQ | 0.963 | 0.108 | Item 4 ~ Group + Module + Sex |
|  | Item 5 ~ Group + Module + Sex + Age + IQ | 0.064 | 0.377 | Item 5 ~ Group + Module + Sex |
|  | Item 6 ~ Group + Module + Sex + Age + IQ | NA | NA | NA |
|  | Item 7 ~ Group + Module + Sex + Age + IQ | 0.746 | 0.479 | Item 7 ~ Group + Module + Sex |
|  | Item 8 ~ Group + Module + Sex + Age + IQ | 0.304 | 0.280 | Item 8 ~ Group + Module + Sex |
|  | Item 9 ~ Group + Module + Sex + Age + IQ | 0.198 | 0.214 | Item 9 ~ Group + Module + Sex |
|  | Item 10 ~ Group + Module + Sex + Age + IQ | 0.538 | 0.417 | Item 10 ~ Group + Module + Sex |
|  | Item 11 ~ Group + Module + Sex + Age + IQ | 0.262 | 0.680 | Item 11 ~ Group + Module + Sex |
|  | Item 12 ~ Group + Module + Sex + Age + IQ | 0.625 | 0.303 | Item 12 ~ Group + Module + Sex |
|  | Item 13 ~ Group + Module + Sex + Age + IQ | 0.693 | 0.234 | Item 13 ~ Group + Module + Sex |
|  | Item 14 ~ Group + Module + Sex + Age + IQ | 0.895 | 0.187 | Item 14 ~ Group + Module + Sex |
|  | Item 15 ~ Group + Module + Sex + Age + IQ | 0.002 | 0.122 | Item 15 ~ Group + Module + Sex + Age |
|  | Item 16 ~ Group + Module + Sex + Age + IQ | 0.137 | 0.762 | Item 16 ~ Group + Module + Sex |
|  | Item 17 ~ Group + Module + Sex + Age + IQ | NA | NA | NA |
|  | Item 18 ~ Group + Module + Sex + Age + IQ | 0.103 | 0.903 | Item 18 ~ Group + Module + Sex |
| ADOS-2, 3q29del without ASD vs. TD | SA CSS ~ Group + Module + Sex + Age + IQ | 0.154 | 0.229 | SA CSS ~ Group + Module + Sex |
|  | RRB CSS ~ Group + Module + Sex + Age + IQ | 0.174 | 0.050 | RRB CSS ~ Group + Module + Sex + IQ |
|  | Item 1 ~ Group + Module + Sex + Age + IQ | 0.047 | 0.516 | Item 1 ~ Group + Module + Sex + Age |
|  | Item 2 ~ Group + Module + Sex + Age + IQ | NA | NA | NA |
|  | Item 3 ~ Group + Module + Sex + Age + IQ | 0.255 | 0.579 | Item 3 ~ Group + Module + Sex |
|  | Item 4 ~ Group + Module + Sex + Age + IQ | 0.055 | 0.369 | Item 4 ~ Group + Module + Sex |
|  | Item 5 ~ Group + Module + Sex + Age + IQ | 0.897 | 0.743 | Item 5 ~ Group + Module + Sex |
|  | Item 6 ~ Group + Module + Sex + Age + IQ | 0.873 | 0.929 | Item 6 ~ Group + Module + Sex |
|  | Item 7 ~ Group + Module + Sex + Age + IQ | 0.996 | 0.298 | Item 7 ~ Group + Module + Sex |
|  | Item 8 ~ Group + Module + Sex + Age + IQ | 0.157 | 0.962 | Item 8 ~ Group + Module + Sex |
|  | Item 9 ~ Group + Module + Sex + Age + IQ | 0.142 | 0.052 | Item 9 ~ Group + Module + Sex |
|  | Item 10 ~ Group + Module + Sex + Age + IQ | 0.084 | 0.992 | Item 10 ~ Group + Module + Sex |
|  | Item 11 ~ Group + Module + Sex + Age + IQ | 0.335 | 0.198 | Item 11 ~ Group + Module + Sex |
|  | Item 12 ~ Group + Module + Sex + Age + IQ | 0.170 | 0.456 | Item 12 ~ Group + Module + Sex |
|  | Item 13 ~ Group + Module + Sex + Age + IQ | 0.077 | 0.935 | Item 13 ~ Group + Module + Sex |
|  | Item 14 ~ Group + Module + Sex + Age + IQ | 0.280 | 0.067 | Item 14 ~ Group + Module + Sex |
|  | Item 15 ~ Group + Module + Sex + Age + IQ | 0.463 | 0.566 | Item 15 ~ Group + Module + Sex |
|  | Item 16 ~ Group + Module + Sex + Age + IQ | 0.056 | 0.094 | Item 16 ~ Group + Module + Sex |
|  | Item 17 ~ Group + Module + Sex + Age + IQ | NA | NA | NA |
|  | Item 18 ~ Group + Module + Sex + Age + IQ | 0.628 | 0.453 | Item 18 ~ Group + Module + Sex |
| ADI-R, 3q29del with ASD vs. nsASD | Domain A ~ Group + Sex + Age + IQ | 0.716 | 0.108 | Domain A ~ Group + Sex |
|  | Domain B ~ Group + Sex + Age + IQ | 0.617 | 0.217 | Domain B ~ Group + Sex |
|  | Domain C ~ Group + Sex + Age + IQ | 0.664 | 0.383 | Domain C ~ Group + Sex |
|  | Domain D ~ Group + Sex + Age + IQ | 0.437 | 0.614 | Domain D ~ Group + Sex |
|  | Sub-domain B1 ~ Group + Sex + Age + IQ | 0.092 | 0.060 | Sub-domain B1 ~ Group + Sex |
|  | Sub-domain B2 ~ Group + Sex + Age + IQ | 0.333 | 0.241 | Sub-domain B2 ~ Group + Sex |
|  | Sub-domain B3 ~ Group + Sex + Age + IQ | 0.215 | 0.406 | Sub-domain B3 ~ Group + Sex |
|  | Sub-domain B4 ~ Group + Sex + Age + IQ | 0.347 | 0.207 | Sub-domain B4 ~ Group + Sex |
|  | Question 42 ~ Group + Sex + Age + IQ | 0.458 | 0.456 | Question 42 ~ Group + Sex |
|  | Question 43 ~ Group + Sex + Age + IQ | 0.182 | 0.121 | Question 43 ~ Group + Sex |
|  | Question 44 ~ Group + Sex + Age + IQ | 0.174 | 0.117 | Question 44 ~ Group + Sex |
|  | Question 45 ~ Group + Sex + Age + IQ | 0.076 | 0.756 | Question 45 ~ Group + Sex |
| ADI-R, relationship between speech delay and nonverbal communication | Domain B ~ Single word + Genotype + Sex + Age + IQ | 0.649 | 0.361 | Domain B ~ Single word + Genotype + Sex |
|  | Sub-domain B1 ~ Single word + Genotype + Sex + Age + IQ | 0.125 | 0.073 | Sub-domain B1 ~ Single word + Genotype + Sex |
|  | Domain B ~ Phrase + Genotype + Sex + Age + IQ | 0.899 | 0.796 | Domain B ~ Phrase + Genotype + Sex |
|  | Sub-domain B1 ~ Phrase + Genotype + Sex + Age + IQ | 0.129 | 0.073 | Sub-domain B1 ~ Phrase + Genotype + Sex |

Goodness of fit analysis to determine whether age and IQ contribute significantly to model fits. Items with NA did not have sufficient variation for regression and were analyzed using Wilcoxon rank sum tests.

**Figures S1. Overlap in nsASD comparators between ADOS-2 and ADI-R.** Venn diagram showing the overlap in nsASD comparators ascertained from NDAR between the ADOS-2 and ADI-R data sets.

**Figure S2. Item-specific performance on the ADOS-2 for individuals with 3q29del and ASD and nsASD comparators.** A-R) ADOS-2 items 1-18, showing the number of participants with 3q29del and ASD and nsASD comparators that scored 0, 1, 2, or 3 on each item.

**Figure S3. Item-specific performance on the ADOS-2 for individuals with 3q29del and no ASD and TD comparators.** A-R) ADOS-2 items 1-18, showing the number of participants with 3q29del and no ASD and TD comparators that scored 0, 1, 2, or 3 on each item.

References

1. Lord C, Risi S, Lambrecht L, Cook EH, Jr., Leventhal BL, DiLavore PC, et al. The autism diagnostic observation schedule-generic: a standard measure of social and communication deficits associated with the spectrum of autism. J Autism Dev Disord. 2000;30(3):205-23.

2. Hus V, Lord C. The autism diagnostic observation schedule, module 4: revised algorithm and standardized severity scores. J Autism Dev Disord. 2014;44(8):1996-2012.

3. Hus V, Gotham K, Lord C. Standardizing ADOS domain scores: separating severity of social affect and restricted and repetitive behaviors. J Autism Dev Disord. 2014;44(10):2400-12.

4. Lord C, Rutter M, Le Couteur A. Autism Diagnostic Interview-Revised: a revised version of a diagnostic interview for caregivers of individuals with possible pervasive developmental disorders. J Autism Dev Disord. 1994;24(5):659-85.

5. Rutter M, Le Couteur A, Lord C. Autism diagnostic interview-revised. Los Angeles, CA: Western Psychological Services. 2003;29(2003):30.
